# Adressing the Value of Checklists in AI Ethics Training: A Response to Kanoshita et al.

**DOI:** 10.1017/jme.2025.10207

**Published:** 2025

**Authors:** Etienne Aucouturier, Alexei Grinbaum

**Affiliations:** 1 Direction des finances et des programmes, Commissariat à l’énergie atomique et aux énergies alternatives Siège administratif, France; 2 Larsim, Commissariat a l’énergie atomique et aux énergies alternatives Direction de la recherche fondamentale, France

Checklists are a structural tool, not a replacement for ethical reflection: Kinoshita et al. rightfully emphasize the valid criticisms already discussed in our paper. Notwithstanding, checklists play a valuable role in promoting ethical reflection. In place of a mere box-ticking approach, our checklist frames a dynamic and adaptive ethical reflection by nudging the user to focus on selecting the most relevant concerns. From a practical and pedagogical standpoint, it also gives trainees a take-home reference document to guide them in their expert work.

Kinoshita et al. seem genuinely puzzled by the proliferation of ethics principles in AI ethics.^1^ We concur. Principles like “explainability” or “non-maleficence,” which form a foundation of digital ethics^2^ and bioethics,^3^ are neither purely descriptive nor purely normative: they are thick concepts that require contextualization. An operationalization of the fundamental principles is never purely a question of design, since ethical judgement about technology involves human desires and emerging preferences, patient needs, and changing norms. Our checklist contains open-ended questions that help to capture all of them in a structured form. As shown during multiple training sessions, the checklist helps the user to quickly identify a broad list of issues: a one-hour session typically suffices to list five to ten questions relevant to a particular use case, whether these are based on the three exercises included in our article or other real-life AI research projects. Thus, the checklist functions as a pedagogical aid, enabling trainees to systematically address the ethical complexities of AI in healthcare within a reasonable time period.

The authors mention ethical questions that do not have a binary yes/no answer and suggest to “quantitatively evaluate and compare the people’s ethical concerns.” We beg to disagree. Once ethics issues have been identified, the more difficult task, which we introduce and discuss in the paper, is to decide on the issues that are “complex or serious,” to use an expression from the EU ethical appraisal scheme.^4^ This is a crucial reflective step, which often requires human dialogue and discussion. A numeric score might help reach consensus, but it must never be the final word: with Buber and Levinas, we stress that ethical judgment must be defined and refined through dialogue.^5^
